# Diagnosis and treatment of the pharyngeal glial choristoma accompanied with incomplete cleft palate: A case report

**DOI:** 10.1097/MD.0000000000013506

**Published:** 2018-12-14

**Authors:** Fang Chen, Hongming Xu, Meizhen Gu, Xiaoyan Li

**Affiliations:** Department of Otorhinolaryngology and Head & Neck Surgery, Shanghai Children's Hospital, Shanghai Jiaotong University, Shanghai, China.

**Keywords:** glia choristoma, incomplete cleft palate, pharyngeal

## Abstract

**Introduction:**

A choristoma formed by heterotopic tissue rarely occurs in the throat, especially one accompanied with cleft palate in a new-born baby.

**Patient concerns:**

An 18-month-old female patient was admitted to the hospital for apparent snoring symptoms accompanied by mouth breathing and sleep apnea. In addition, the patient presented with weak aspiration and nasal leakage during fluid intake.

**Diagnosis:**

The patient received routine physical examination and endoscopy showing that there was a wide fissure which split from the palate vertical anterior cleft to 1/3 of the hard palate. Meanwhile, we found an unclear-bordered uplift in the left palate and a soft mass. The radiographs revealed a mass with inhomogeneous density convex to the pharyngeal cavity.

**Interventions:**

The patient was subsequently referred for surgical resection and tissue diagnosis of choristoma was confirmed by pathological examination. H&E staining showed well demarcated mature brain tissue with scattered sand-like calcification.

**Outcomes:**

According to the diagnosis, the patient suffered from pharyngeal glial choristoma and incomplete cleft palate. The surgical resection and repair were performed together. The postoperative recovery was very good.

**Lessons:**

Choristoma rarely occurs in the head and neck, especially if accompanied by cleft palate. Early diagnosis for choristoma relies heavily on clinical examination and radiological imaging. Complete resection of choristoma remains the gold standard for treatment of these patients.

## 1 Introduction

Glial choristoma, also named heterotopic central nervous system tissue, is a benign tumor involving with heterotopic tissue.^[1]^ The most common histological types are epidermoid cysts and dermoid tumors. Simple and complex choristomas are relatively rare. There are reports of choristomas encountered in the eye, tongue, pituitary gland, stomach, liver, adrenal gland as well as in the case of endometriosis in the pelvis. However, it rarely occurs in the throat especially when accompanied by a cleft palate.^[2–4]^ As with many other types of choristomas, offering the most the appropriate management relies heavily on radiological imaging, and when possible, histological diagnosis from a diagnostic biopsy. Currently, the gold standard management for pharyngeal glial choristoma is complete surgical excision. Here, we presented a case of an 18-month-old baby girl with pharyngeal glial choristoma accompanied by cleft palate. We then provided pharyngeal resection and cleft palate repair with consent from the parents. The patient recovered well after surgery. The present study may increase the clinical knowledge of this disease and help physicians appropriately diagnose and manage it.

## 2 Case report

An 18-month-old female patient was admitted to Shanghai Children's Hospital (Shanghai, China) for appearant snoring symptoms accompanied by mouth breathing and sleep apnea. In addition, the patient presented with weak aspiration and nasal leakage during fluid intake.

**Figure 1:**
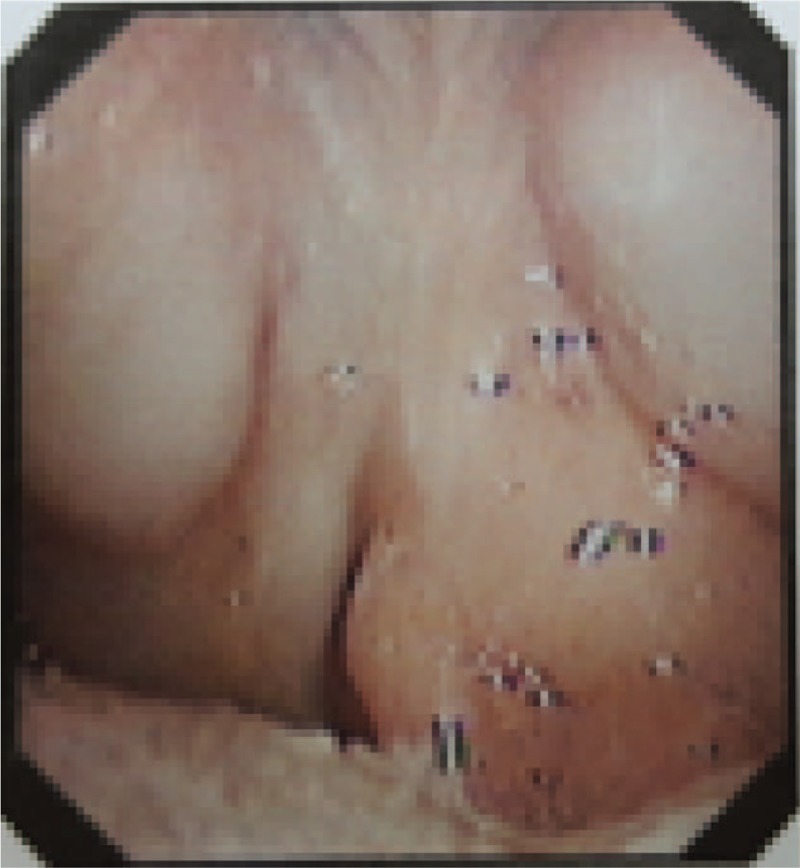
Preoperative view of pharyngeal: left palate tumor and incomplete cleft palate.

**Figure 2:**
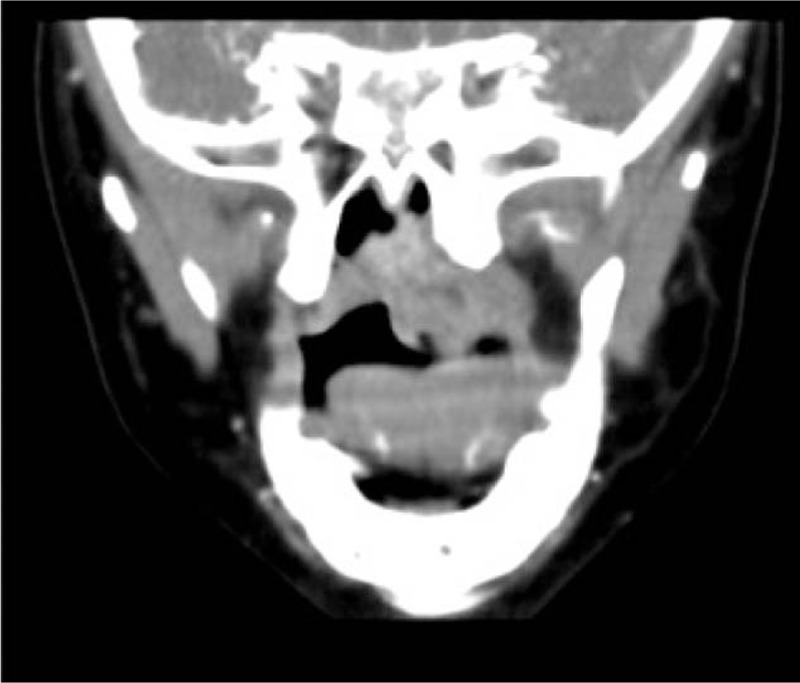
Coronal CT image showed a mass convex to the pharyngeal cavity (about 27 mm × 21 mm × 26 mm in size). CT = computed tomography.

The routine physical examinations showed a wide fissure which split from the palate vertical anterior cleft to 1/3 of the hard palate. There was no congestion in pharyngeal mucosa with the uvula in the middle. Bilateral tonsils showed hypertrophy without exudation. Epiglottis and bilateral pear-like nest were not clear. The pear bone could be seen. Thus, the patient had incomplete cleft palate. Meanwhile, we also found an unclear-bordered uplift in the left palate and a soft mass which could be moved in the right soft palate trailing edge with endoscopy (Fig. 1). Then, the patient underwent a computed tomography (CT) scan. Consistently, radiographs of the palate revealed a mass convex to the pharyngeal cavity which was about 27 mm × 21 mm × 26 mm in size (Fig. 2). In addition, no obvious abnormality was found on the nasopharyngeal bone or soft tissue. According to the above examination, the diagnosis for the patient was incomplete cleft palate and soft palate vascularized space-occupying lesions (involving the left posterior wall of the nasopharynx, most likely fibrous hemangioma).

**Figure 3:**
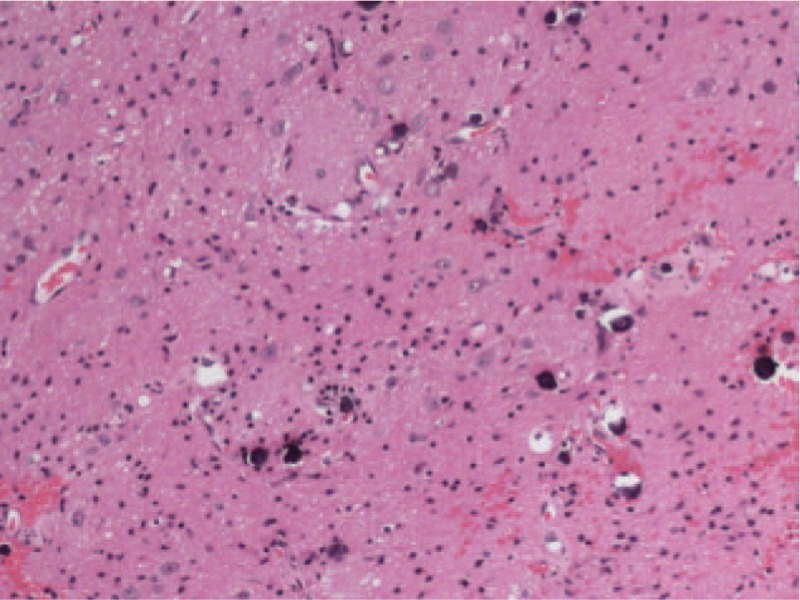
Histological examination showing a well demarcated mature brain tissue with scattered sand-like calcification (hematoxylin and eosin, original magnification 400 × ).

The patient was treated by palatal lump resection and cleft palate repair with endoscopic guidance from the oral cavity while under general anesthesia. During the surgical resection process, the mass was dissected from the dorsal side of the left soft palate with the pharyngeal mucosa preserved and the nasopharyngeal mucosa completely removed. At the end of the surgery, the plasma radiofrequency knife was used to ablate the residual tumor tissue. After proper hemostasis, the cleft palate was also repaired. Postoperative symptomatic treatment of infection was done with nasal feeding. Histological examination showed that there was a well-demarcated mature brain tissue with scattered sand-like calcification (Fig. 3).

The patient had an excellent recovery. And one month follow up with endoscopy showed good wound healing without rupture. The snoring, mouth breathing, and sleep apnea symptoms disappeared. Also, the patient no longer experiences nasal leakage with fluid intake.

## 3 Discussion

Pharyngeal glial choristoma is a rare congenital non-neoplastic disease caused by abnormal development of the central nervous system.^[5,6]^ Most choristomas occur in the head and neck and commonly involve the nose,^[7,8]^ tongue^[9,10]^ or middle ear^[11,12]^. Radiographic examination could determine the location and extent of lesions. Most importantly, axial and coronal CT images of the head are very useful in determining the location of the tumor and its relation to the skull base, especially for those located in the cranium. For patients with lesions involving the nasopharynx, clinicians should discern these glial choristomas from other diseases including brain meningocele, lymphangioma, teratoma, ectopic thyroid glioblastoma among others. The gold standard for diagnosis of choristomas is pathological examination. Glial choristomas generally occur at birth or in young people. Most glial choristomas grow slowly at a rate parallel to the surrounding tissue. As a result, patients who suffer this disease usually have no complaints of discomfort. Surgical resection is an effective treatment. The postoperative prognosis for these patients is generally good, and no malignant transformation or recurrence has been reported. Thus, early neonatal surgery is still a necessary and urgent therapy which can prevent bone deformation and facial deformity caused by further growth of a glioma and is conducive to normal swallowing function and the development of pharyngeal coordination.^[13]^

Incomplete cleft palate means a complete cleft soft palate accompanied by incomplete cleft hard palate.^[14]^ But the alveolar is complete. The real etiology is still unknown, but environmental and genetic factors are involved in this pathology. These patients suffer from physiological development defects, maxillofacial shape secondary changes, surgical trauma, language, hearing, and other dysfunction, which decreases a patient's quality of life. Most importantly, it brings serious psychological trauma for both the patients and their families. The current cleft palate repair technology mainly takes palatine saphenous muscle function reparation or the soft palate extend as a starting point, to restore children's eating and speech function.^[15]^ And, it is believed that surgical intervention is necessary to guarantee adequate growth and functional development. Patients obtain the best results through the combination of surgical and orthodontic treatment.^[16]^

To this day, the exact mechanisms of this disease are still unknown. Two potential hypotheses have been generated regarding the pathogenesis. One hypothesis supposes that protrusion of neural glial tissue from developing cerebral tissue becomes isolated from the brain during later development. It has been reported that most head and neck choristomas might develop in this way. The other hypothesis regards the displacement of neuroectodermal cells at early stage of embryogenesis, which differentiaes into neurogial tissue in ectopic sites.^[5,17,18]^ However, the detailed mechanisms for the development of this disease still needs to be investigated in the future.

To our best knowledge, there are no case reports about choristoma accompanied by incomplete cleft palate. However, based on published data and our clinical experiences, the appropriate management for the patients should be complete resection of the pharyngeal mass and cleft palate repair at the same time. The patient had an excellent recovery from surgery, consistent with published data. Taken together, pharyngeal resection combined with cleft palate repair is a necessary and efficient treatment for children suffering from pharyngeal glial choristoma accompanied by incomplete cleft palate.

## Author contributions

**Conceptualization:** Fang Chen, Meizhen Gu, Xiaoyan Li.

**Data curation:** Fang Chen, Hongming Xu, Meizhen Gu, Xiaoyan Li.

**Formal analysis:** Fang Chen, Hongming Xu, Meizhen Gu, Xiaoyan Li.

**Funding acquisition:** Meizhen Gu, Xiaoyan Li.

**Investigation:** Hongming Xu, Meizhen Gu, Xiaoyan Li.

**Methodology:** Fang Chen, Hongming Xu, Meizhen Gu, Xiaoyan Li.

**Project administration:** Fang Chen, Meizhen Gu, Xiaoyan Li.

**Resources:** Fang Chen, Hongming Xu, Meizhen Gu, Xiaoyan Li.

**Software:** Fang Chen, Hongming Xu, Meizhen Gu, Xiaoyan Li.

**Supervision:** Fang Chen, Meizhen Gu, Xiaoyan Li.

**Validation:** Fang Chen, Hongming Xu, Meizhen Gu, Xiaoyan Li.

**Visualization:** Fang Chen, Hongming Xu, Meizhen Gu, Xiaoyan Li.

**Writing – original draft:** Fang Chen, Hongming Xu, Meizhen Gu, Xiaoyan Li.

**Writing – review & editing:** Fang Chen, Hongming Xu, Meizhen Gu, Xiaoyan Li.
